# Adsorption Behavior of NO and NO_2_ on Two-Dimensional As, Sb, and Bi Materials: First-Principles Insights

**DOI:** 10.3390/ma17051024

**Published:** 2024-02-23

**Authors:** Yuting Zhang, Xi Chen, Dan Fang, Hao Yan, Dengkui Wang, Xiaohua Wang, Jinhua Li, Yingjiao Zhai, Xueying Chu, Dongbo Wang, Hongbin Zhao, Xuan Fang

**Affiliations:** 1 State Key Laboratory of High Power Semiconductor Lasers, School of Physics, Changchun University of Science and Technology, 7089 Wei-Xing Road, Changchun 130022, China; 2021100148@mails.cust.edu.cn (Y.Z.); 18843111982@163.com (X.C.); wccwss@foxmail.com (D.W.); biewang2001@126.com (X.W.); jhli_cust@163.com (J.L.); yjzhai@cust.edu.cn (Y.Z.); xueying_chu@cust.edu.cn (X.C.); zhaohongbin@grinm.com (H.Z.); fangx@cust.edu.cn (X.F.); 2 Department of Opto-Electronic Information Science, School of Materials Science and Engineering, Harbin Institute of Technology, Harbin 150001, China; wangdongbo@hit.edu.cn; 3 State Key Laboratory of Advanced Materials for Smart Sensing, General Research Institute for Nonferrous Metals, Beijing 100088, China

**Keywords:** two-dimensional materials, first-principles computations, constituent materials, gas adsorption

## Abstract

To address the most significant environmental challenges, the quest for high-performance gas sensing materials is crucial. Among numerous two-dimensional materials, this study investigates the gas-sensitive capabilities of monolayer As, Sb, and Bi materials. To compare the gas detection abilities of these three materials, we employ first-principles calculations to comprehensively study the adsorption behavior of NO and NO_2_ gas molecules on the material surfaces. The results indicate that monolayer Bi material exhibits reasonable adsorption distances, substantial adsorption energies, and significant charge transfer for both NO and NO_2_ gases. Therefore, among the materials studied, it demonstrates the best gas detection capability. Furthermore, monolayer As and Sb materials exhibit remarkably high capacities for adsorbing NO and NO_2_ gas molecules, firmly interacting with the gas molecules. Gas adsorption induces changes in the material’s work function, suggesting the potential application of these two materials as catalysts.

## 1. Introduction

With the acceleration of industrialization and urbanization, the issue of air pollution has gained significant prominence [1,2]. Pollutant gases not only jeopardize natural ecosystems but also pose a considerable threat to human health [3,4,5]. Studies have demonstrated the connection between air pollution and kidney function as well as potential regulatory mechanisms, and an increasing body of research is connecting air pollution to the health of several organs in the human body. Research on the effect of air pollution on labor productivity has been performed by several academics. In other words, more people are becoming aware of harmful gasses. Up to now, various gas detection methods have been established, such as electrochemical, chromatography, infrared spectroscopy, and spectrophotometry. However, the large-scale applications of these methods are still limited by their complicated operation, high cost, or inability to detect in real-time [6]. Nitrogen oxides (NO_x_ = NO + NO_2_) emitted from stationary combustion chambers (including waste to energy plants) or engines cause numerous undesirable environmental effects. These include negative influences on human and animal health, detrimental effects on plants and vegetation, acid rain, and smog. These negative influences are widely recognized by the scientific community. Nitric oxide (NO) and Nitrogen dioxide (NO_2_) are prominent atmospheric pollutants [7,8,9], making the monitoring and regulation of their emissions a pivotal concern [10]. Consequently, there is a growing need for nitrogen oxide gas detectors characterized by high sensitivity, selectivity and stability. Various methods of NO_X_ detection have been published, including electrochemical gas sensors [11,12,13], optical gas sensors [14], and semiconductor gas sensors [15,16,17]. Compared with alternative gas sensors, semiconductor gas detectors display heightened sensitivity and affordability. This is due to the semiconductor materials utilized having excellent electron mobility and high carrier concentration, which cause the gas to interact with the semiconductor and generate electrons and holes, changing the electric current and allowing for the detection of gas [18]. However, these detectors are not without constraints, requiring improved selectivity and stability, and there is an urgent need to identify superior gas sensor materials [19].

Two-dimensional materials, owing to their substantial body surface ratio, abundant active sites, and controllable morphology, have emerged as highly promising candidates for gas sensing applications [20,21,22,23,24,25]. Following the successful detection of individual NOx gas molecules by graphene [26], the adsorption characteristics of NOx molecules onto other two-dimensional materials have also been extensively investigated. For example, in the research conducted by Ta, Q. T. H., by employing a combined approach of high-temperature Si diffusion and HF corrosion, an in situ synthesis of Si@TiO_2_/Ti_3_C_2_T_X_ heterogeneous structures based on the Ti_3_AlC_2_ MAX phase was achieved. Through Density Functional Theory (DFT) calculations, it was discovered that Si atoms may enhance the NO_2_ adsorption process. The findings of this study may validate the potential of Si@TiO_2_/Ti_3_C_2_Tx heterogeneous structures as multi-functional nanomaterials [27]. In extensive studies, materials containing Group V elements have demonstrated outstanding gas detection capabilities [28,29,30,31]. For example, Ou et al. investigated the sensing capacity of single-walled black phosphorus nanotubes (BPNTs) for a number of typical harmful gas compounds using ab initio density functional theory. Analysis of charge density difference and band structure indicated the electronic properties of BPNTs are significantly altered after the adsorption of NO_2_ [28]. In addition, Ye et al. applied violet phosphorene to gas sensing for the first time. The bulk violet phosphorus was fabricated by chemical vapor transport and exfoliated by a tap method and ultrasonic method to violet phosphorene. It was then combined with graphene for sensing NO and CO gas molecules [29]. In Yang, L.’s work, lateral heterojunctions (LHSs) based on arsenic and antimony were designed along the armchair (AC) or zigzag (ZZ) edges. The adsorption and sensing characteristics of As/Sb LHSs to NO_2_ before and after applying different types of strain were calculated by first principles. This work laid the foundation for the fabrication of high-performance NO_2_ gas sensors [32]. Earlier investigations demonstrated that monolayer black phosphorus exhibits superior chemical sensing capabilities for NO_2_ and NO, with comparable or even higher molecular adsorption energies compared with prominent two-dimensional materials like graphene or MoS_2_. However, black phosphorus has an extremely low air stability, making it vulnerable to disintegration after only a few days of exposure to normal ambient conditions [33,34]. The creation of gas detectors based on black phosphorus is significantly hampered and challenged by this inherent instability. Antimonene has also shown potential for gas adsorption, but the study of this material is not comprehensive enough [35,36]. Considering the significant gas sensing potential of group V monolayer materials, a comprehensive investigation of the gas adsorption properties of monolayer pentaenes is warranted.

In this study, our objective is to compare the gas sensing capabilities of monolayer arsenic (As), antimony (Sb), and bismuth (Bi) towards nitrogen oxides (NO_X_). Utilizing first-principle calculations, we established a monolayer material model to investigate the adsorption behavior of NO and NO_2_ on monolayer As, Sb, and Bi materials. It is noted that monolayer Bi exhibits the potential for NO_X_ gas detection. We systematically examined the adsorption energies, density of states (DOS), and work functions of As, Sb, and Bi materials for NO and NO_2_ gases. A comprehensive assessment of the gas detection applicability of these three materials was conducted from the perspectives of adsorption energy and distance. The results indicate that monolayer antimony (Sb) demonstrates the optimal gas sensing performance, showing the maximum adsorption distance, significant adsorption energy, and charge transfer during the adsorption of NO_2_ and NO. Conversely, monolayer arsenic (As) and monolayer antimony (Sb) exhibit similarly excellent adsorption energies and charge transfers for NO_2_ and NO, but the adsorption behavior is characterized by molecules being too close to the material surface, possibly indicating overly intense adsorption unsuitable for gas sensing. However, due to strong interactions between the materials and gases, as well as the tunable work functions within a small range, monolayer arsenic and antimony hold potential as catalyst materials.

## 2. Computational Details

First-principles density functional theory (DFT) was performed based on the Vienna Ab initio Simulation Package (VASP) [37]. Generalized gradient approximation (GGA) with the Perdew–Burke–Ernzerhof (PBE) [38] functional was employed to deal with the exchange–correlation interactions of electrons. The Electron–ion interactions were described using the projector augmented wave (PAW) [39] potentials. The van der Waals (vdW) effects were included using D3-Grimme [40,41] correction to obtain better long-range van der Waals interaction between atomic layers. The cutoff energy for the plane-wave basis was 600 eV. The Brillouin zone of the surface unit cell was sampled by Monkhorst–Pack (MP) grids, with a k-point mesh density of 2π × 0.04 Å^−1^ for structures optimizations [42]. A vacuum layer of 15 Å was introduced to avoid interactions between periodic images. Geometry optimization was performed under the condition that the total energy convergence was less than 10^−6^ eV and the atomic force was less than 0.01 eV/Å. The density of states (DOS) of the As, Sb and Bi monolayers were calculated using the PBE functional.

In our calculations, the adsorption capacity of a monolayer for gas molecules can be expressed using the adsorption energy *E_ad_* as follows:

$$E_{ad}=E_{\mathrm{Mat}+\mathrm{Gas}\;}-E_{\mathrm{Mat}\;}-E_{\mathrm{Gas}\;}$$

where *E*_Mat+Gas_ represents the total energy of a gas molecule adsorbed in the monolayer system, and *E*_Mat_ and *E*_Gas_ represent the energy of the monolayer and free gas molecule, respectively. The larger the absolute value of *E_ad_*, the stronger the adsorption capacity. Moreover, the Bader charge was used to analyze the charge transfer between the monolayer and gas molecule [43].

## 3. Results and Discussion

**Figure 1:**
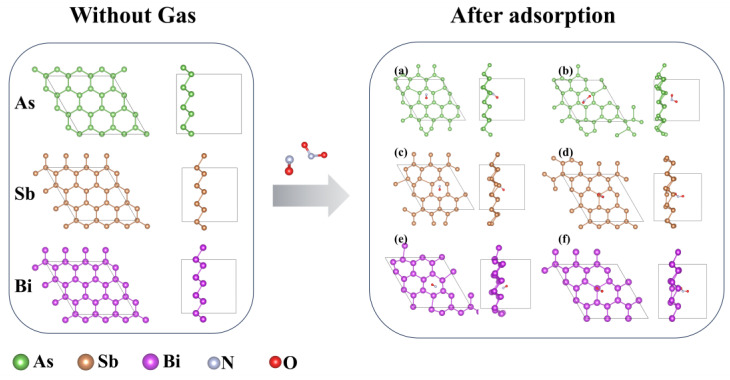
Stable adsorption structure of NO molecule on (**a**) monolayer As, (**c**) monolayer Sb and (**e**) monolayer Bi; stable adsorption structure of NO_2_ molecule on (**b**) monolayer As, (**d**) monolayer Sb and (**f**) monolayer Bi.

The original monolayer As, Sb, and Bi materials exhibit regular hexagonal structures. The calculated lattice constants for the monolayers As, Sb, and Bi are 3.81 Å, 4.38 Å, and 4.58 Å, respectively. In our previous research within the research group, we evaluated the stability of all structures involved from the perspective of formation energy, demonstrating that the structures can exist spontaneously in a stable manner [44,45,46]. After optimization calculation, we found that the most stable adsorption sites of NO and NO_2_ molecules on these three materials were located above the interface. In the case of NO gas adsorption, the NO molecules adopt an inclined structure above the monolayer material, with N atoms positioned close to the material surface. Similarly, for NO_2_ gas adsorption, the NO_2_ molecules assume an inclined structure above the monolayer material, with O atoms positioned in proximity to the material. The As, Sb, and Bi monolayer materials’ structural diagrams, both before and after gas adsorption, are displayed in Figure 1. The adsorbed structure is on the right side, and the unadsorbed structure is on the left. The outlined boxes on the material in the figure represent individual unit cells, with a unit cell size of 4 × 4 × 1. It is evident that the As, Sb, and Bi monolayer materials exhibit a regular hexagon with no deformation and a regular structure when no gas is adsorbed. The materials’ bond lengths alter to varying degrees after adsorbing gas, and the substance stops exhibiting Periodic regular hexagons. Both the top and side views clearly show the changes in material shape. The presence of charge transfer, indicated by the change in bond length, suggests that there is clear adsorption behavior between the material and the gas.

**Figure 2:**
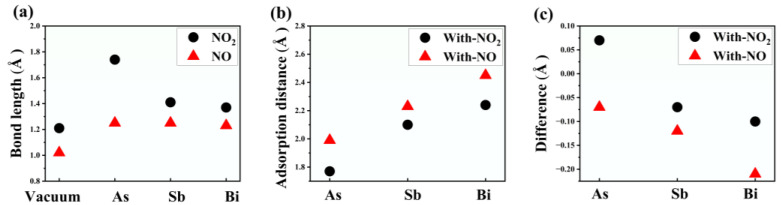
The (**a**) bond length and (**b**) adsorption distance of NO and NO_2_ gases adsorbed on monolayers of As, Sb, and Bi; (**c**) The difference between the sum of the covalent atomic radii and the adsorption distance of NO and NO_2_ gases adsorbed on monolayers of As, Sb, and Bi.

We have defined the adsorption distance as the minimum distance between the atoms in gas molecules and the atoms in monolayer materials during the adsorption process. To optimize the geometry, we initially obtained N-O bond lengths of 1.17 Å and 1.21 Å for free NO and NO_2_ molecules, respectively. However, upon adsorption of NO molecules onto the three monolayer materials, we observed an elongation in the N-O bond lengths. It is commonly accepted that the sum of two covalent atomic radii represents the ionic bond length. Previous studies [47] have provided atomic covalent bond lengths. By subtracting the adsorption distance from the sum of the covalent atomic radii, we can assess the strength of adsorption and compare the adsorption capacities of different materials. These data are summarized in Figure 2.

The deformation of the material illustrated in Figure 1 can be explained by examining the changes in bond length data shown in Figure 2a and the adsorption distance depicted in Figure 2b. The extent of bond length change and adsorption distance can determine the occurrence of adsorption. As depicted in Figure 2c, when the monolayer As material adsorbs NO_2_, the adsorption distance is smaller than the sum of the covalent atomic radii, this indicates that the adsorption capacity is extremely strong, which is unfavorable for desorption and is not suitable for gas detection. In the other adsorption cases (As-NO, Sb-NO, Sb-NO_2_, Bi-NO, Bi-NO_2_), the adsorption distance is slightly larger than the sum of the covalent atomic radii, indicating that the materials are sensitive to gas and possess moderate adsorption strength. The difference between the sum of the covalent atomic radii and the adsorption distance when monolayer As, Sb, and Bi materials adsorb the two gases follows a clear decreasing trend. For NO_2_ adsorption (0.07 Å, −0.07 Å, −0.1 Å) and NO adsorption (−0.07 Å, −0.12 Å, −0.23 Å), the bond energy gradually decreases, resulting in a reduction in the adsorption capacity of arsenene, antimonene, and bismuthene for the gases.

To investigate the gas sensing characteristics of As, Sb, and Bi monolayer materials further, Bader analysis was used to determine the charge transfer (ΔQ) between the materials and NO and NO_2_ gas molecules, as indicated in Table 1. All adsorption systems with two gases and three materials have negative computed charge transfer quantities. When NO_2_ gas is adsorbed on As, Sb and Bi monolayer materials, the materials donate electrons to the NO_2_ molecules, while N atoms donate electrons to the O atoms. The substance serves as a charge donor, and the NO_2_ gas serves as a charge acceptor throughout the charge transfer process; electrons from As, Sb, and Bi monolayer materials are transferred to N and O atoms when they adsorb NO gas. In the charge transfer process, the gas serves as an acceptor, and the electrons are moved from the substance to the molecules of NO gas. Charge accumulation results from the material’s continued role as a charge donor and the NO gas’s role as an acceptor. As a result, it is possible to compare the acceptor doping process to the adsorption of NO and NO_2_ molecules. These results are in line with the elongation of the N-O bond length during the adsorption of the two gases, as depicted in Figure 2.

**Figure 3:**
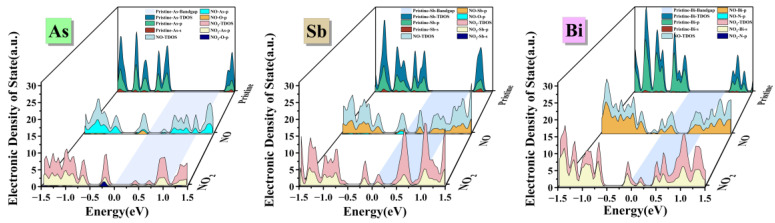
Total density of states and partial density of states of gas molecules on monolayers As, Sb and Bi.

We drew a DOS (Density of States) distribution map and examined the changes in the material’s electrical characteristics before and after adsorption to have a better understanding of the possible adsorption mechanism. The E_f_ (Fermi level) is set at energy 0 in the DOS calculation. Figure 3 displays, from left to right, the DOS and PDOS for As, Sb, and Bi monolayer materials under various adsorption circumstances. Three adsorption situations are distinguished for each material: unadsorbed, adsorbed NO, and adsorbed NO_2_. The patches with varying colors and heights beneath the TDOS indicate how each orbital contributes to the TDOS under various adsorption circumstances. A blue region that stretches from the material’s DOS when it has not adsorbed gas to its DOS after it has is shown at the bottom of each image. It is simple to compare the bandgaps of the material before and after adsorption since this region shows the bandgap width of the material when gas has not been adsorbed. Similar changes can be seen when comparing the TDOS of As, Sb, and Bi monolayer materials before and after adsorption. First, the blue area at the bottom of the picture makes it evident that, following adsorption under various adsorption conditions, there are essentially new energy states on the bandgap, and the bandgap width varies in comparison with the unadsorbed gas. This demonstrates that energy exchange takes place between the material and gas during the adsorption process, changing the substance’s electrical characteristics; moreover, the height of the peak DOS following gas adsorption is lower than when the gas is not adsorbed. This indicates that the system releases energy outward during the adsorption process, resulting in a decrease in system energy after gas adsorption. This is in line with the findings computed in Table 1: during the adsorption process, heat is produced and charge is transferred from the substance to the gas. This indicates that the study’s adsorption mechanism is rather likely to occur.

The As monolayer material in Figure 3 (As) has an initial bandgap width of 0.62 eV, and the E_f_ in DOS is situated close to the E_V_. The substance displays characteristics of a P-type semiconductor. According to the PDOS study, the As-p orbital (the p orbital of As atoms) contributes most to the material’s DOS intensity, whereas the As-s orbital (the s orbital of As atoms) contributes comparatively less. As-p and O-p orbitals at the top of the E_a_, the E_f_ moving toward the E_v_, and the appearance of a new energy level at the bandgap all contribute to the DOS of the material system in the adsorption system generated by the material adsorbing NO gas. This is because the material’s electrons move toward NO molecules during adsorption, increasing the material’s hole concentration. Similar to this, when the material adsorbs NO_2_ gas, both As-p and O-p orbitals contribute to the DOS, and the E_f_ moves towards the E_v_. The material demonstrates semiconductor characteristics in these two adsorption scenarios due to the orbital hybridization phenomena, in which the E_f_ does not cross the E_v_.

The E_f_ is situated close to the E_v_ in Figure 3 (Sb), the DOS diagram of the Sb monolayer material’s initial state, with a bandgap width of 0.48 eV. The substance displays characteristics of a P-type semiconductor. Sb-p orbitals contribute more to a material’s DOS strength than Sb-s orbitals do. Orbital hybridization between Sb-p and O-p orbitals occurs in the adsorption system generated by the material adsorbing NO, resulting in a change in the DOS to the left. There are smaller energy levels at the E_f_, which is located at the bottom of the conduction band. The substance has a modest metallicity. When adsorbing NO_2_, there was no discernible orbital hybridization event, and the Sb-p orbital continued to contribute the majority of DOS. The appearance of an energy state at the E_f_ in these two adsorption scenarios shows how adsorption affects the material’s electrical characteristics.

The E_f_ is situated close to the E_v_ in Figure 3 (Bi), the DOS diagram of the initial state of the Bi monolayer material, with a bandgap width of 0.89 eV. The substance displays characteristics of a P-type semiconductor. PDOS analysis reveals that the primary source of DOS intensity is the Bi-p orbitals, with the Bi-s orbitals contributing comparatively little. The E_f_ moves to the left and crosses the top of the E_v_ in the adsorption system created by the material adsorption of NO gas. At the E_f_, a higher energy state emerges, and orbital hybridization is feeble. Bi-p orbitals contribute the most to the DOS intensity, followed by N-p orbitals. The bottom of the conduction band passes the E_f_ and the DOS peak moves to the left when the material adsorbs NO_2_. A new, weak energy state emerges at the E_f_ as a result of the electron exchange with the gas molecule. In PDOS, this orbital hybridization is particularly weak, Bi-s orbitals account for the majority of the DOS intensity, with N-p orbitals contributing very little. This suggests that NO_2_ gas has minimal impact on Bi monolayer materials’ electrical characteristics.

It is evident from the three materials’ performances in adsorbing gas that As and Sb monolayer materials significantly affect DOS while adsorbing gas. A strong orbital hybridization phenomenon can be observed in the DOS graph after adsorption, indicating the strong sensitivity of As and Sb monolayer materials to NO and NO_2_ gases. The height, position, and bandgap width of the DOS peak all show significant changes. Nevertheless, Bi monolayer materials show comparable significant changes in the DOS plot upon gas adsorption. One notable distinction is that the adsorbed PDOS exhibits weaker orbital hybridization phenomena, and the material maintains gas sensitivity without an unduly high gas binding.

To explore the sensing performance of the three materials, the work function (WF) was calculated under all adsorption conditions. The work function represents the minimum energy required to remove electrons from the surface of a solid material and is expressed as *Φ = E_vac_ − E_f_*, where *E_vac_* and *E_f_* are the electrostatic potentials of the electron far from the surface and at the Fermi level, respectively [48,49,50]. *E_vac_* is defined as 0.

**Figure 4:**
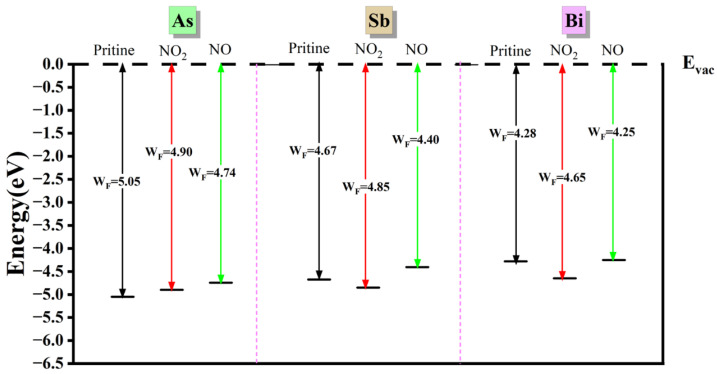
Calculated work functions of pristine monolayer and gas molecules adsorbed on the substrate monolayers As, Sb and Bi.

In Figure 4, the work functions of monolayer As, Sb, and Bi materials before and after adsorbing NO_2_ and NO are summarized. From left to right, the work functions of the As, Sb, and Bi monolayer materials are computed. The original state without gas adsorption, the work function following NO adsorption, and the work function following NO_2_ adsorption are the three adsorption states for which the work function values for each material are computed. It can be observed that different gas adsorption leads to adjustable work functions within the ranges of 4.74–5.05 eV, 4.40–4.85 eV, and 4.25–4.65 eV for monolayer As, Sb, and Bi materials, respectively. Notably, the work functions of all three materials show a gradual decrease even when not adsorbed, with values of 5.05 eV, 4.67 eV, and 4.28 eV, respectively. Upon gas adsorption, the work functions exhibit a similar pattern with a decrease in magnitude. The Bi-NO work function shows minimal change, with only a 0.03 eV difference, suggesting that gas adsorption can modulate the work functions of monolayer As, Sb, and Bi materials within a certain range.

The decrease in work function corresponds to an increase in the Fermi energy level, which can enhance the electron transfer rate at the material interface and improve their catalytic performance. Remarkably, monolayer As and Sb materials exhibited a significant decrease in work function after gas adsorption, indicating their potential application in catalysis [51].

**Figure 5:**
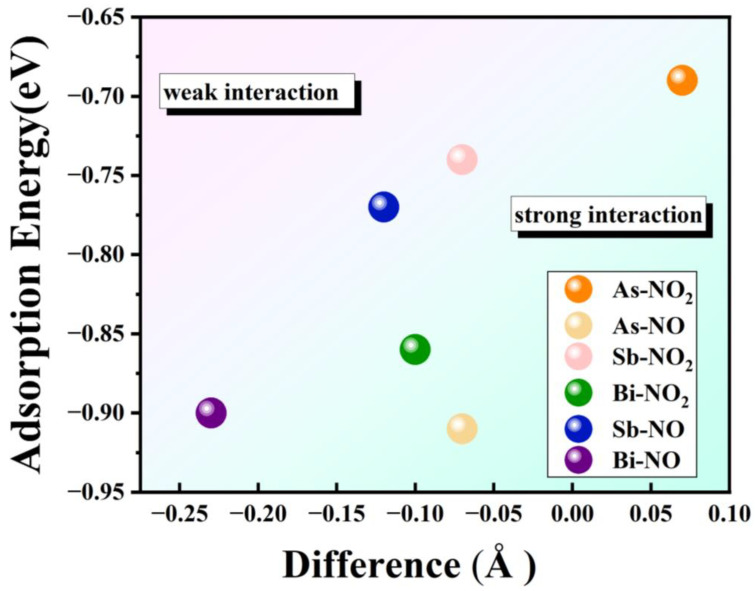
The relationship between adsorption distance and adsorption energy between NO and NO_2_ molecules and substrate monolayers As, Sb and Bi.

The strength of gas molecule adsorption on solid surfaces is determined by the absolute value of the adsorption energy and the size of the adsorption distance [52,53]. Generally, a larger absolute value of adsorption energy and a smaller adsorption distance indicate a stronger interaction between gas molecules and solid surfaces, resulting in a more stable adsorption phenomenon. Conversely, a smaller absolute value of adsorption energy and a larger adsorption distance suggest a weak interaction between gas molecules and the solid surface, leading to an unstable adsorption phenomenon and a higher likelihood of desorption reactions. The difference between the adsorption energy under different adsorption conditions and the sum of the adsorption distance and covalent atomic radius is summarized in Figure 5. The larger the difference, the tighter the adsorption and the closer the gas molecules are to the substance following adsorption. The adsorption strength increases with the absolute value of the adsorption energy. As a result, the evaluation criteria of difference and adsorption energy divide the adsorption scenario in Figure 5 into two categories: weak interaction and strong interaction.

We have listed the adsorption energies under different adsorption scenarios in Table 2. It can be observed that all three materials exhibit negative adsorption energies when adsorbing NO and NO_2_ gases, indicating heat release during the adsorption process. This aligns with the electron transfer from the material to gas molecules described in Table 1, where significant charge transfer and large absolute values of adsorption energy are observed. These findings suggest a propensity for adsorption in all studied cases. Monolayer Bi material demonstrates high adsorption energy and moderate adsorption distance when adsorbing NO and NO_2_ gases, indicating its potential for effective gas sensing detection. Although the adsorption distance of monolayer As and monolayer Sb materials is slightly greater than the sum of the covalent atomic radius when adsorbing NO and NO_2_ gas, they still exhibit strong gas adsorption capacities, which makes desorption less likely and renders them less suitable for gas sensing detection.

## 4. Conclusions

In summary, we have constructed a model of monolayer As, Sb and Bi materials and investigated their gas sensing performance towards NO and NO_2_ gases using first-principles calculations. The results reveal that the monolayer Bi material exhibits a moderate adsorption distance for both NO and NO_2_ gases, accompanied by a significant adsorption energy and charge transfer. This indicates its excellent gas sensing ability. Upon gas molecule adsorption, the material undergoes noticeable deformation, and strong orbital hybridization occurs between the gas and the material. New energy states emerge within the bandgap, highlighting the strong interaction between the monolayer Bi material and gas molecules. In contrast, the monolayer As and Sb materials react strongly to NO and NO_2_ gases. They also exhibit narrow adsorption distances, high adsorption energy, and charge transfer during adsorption. Following adsorption, there is significant material deformation, evident bond length alterations, considerable orbital hybridization between the material and gas, and the appearance of several new energy states at the band gap. The monolayer As and Sb materials exhibit very small adsorption distances with NO and NO_2_ gas molecules, indicating an overly strong adsorption capacity that hinders desorption and renders them less suitable for gas sensing detection. However, considering their strong interaction with the gas molecules and the ability to slightly modulate the work function, they hold potential as catalyst materials.

## Figures and Tables

**Table 1:** Calculation of charge transfer adsorbed by gas molecules on monolayer materials.

Adsorption Type	Material	N	O	Charge Transfer (C)	Effect
As-NO	0.79	−0.31	−0.47	−0.79	Acceptor
As-NO_2_	0.89	0.37	−1.25	−0.89	Acceptor
Sb-NO	0.82	−0.25	−0.57	−0.82	Acceptor
Sb-NO_2_	0.65	0.45	−1.1	−0.65	Acceptor
Bi-NO	0.58	−0.56	−0.02	−0.58	Acceptor
Bi-NO_2_	0.66	0.4	−1.06	−0.66	Acceptor

**Table 2:** Adsorption energy values under various scenarios.

Adsorption Type	Adsorption Energy (eV)
As-NO	−0.91
As-NO_2_	−0.69
Sb-NO	−0.77
Sb-NO_2_	−0.74
Bi-NO	−0.9
Bi-NO_2_	−0.86

## Data Availability

Data are contained within the article.
